# Editorial: DNA Replication Stress and Cell Fate

**DOI:** 10.3389/fcell.2021.778486

**Published:** 2021-10-25

**Authors:** Lin Deng, Chun-Long Chen, Yuanliang Zhai, Yunzhou Dong, Huiqiang Lou

**Affiliations:** ^1^Shenzhen Bay Laboratory, Shenzhen, China; ^2^Institut Curie, PSL Research University, CNRS UMR3244, Dynamics of Genetic Information, Sorbonne Université, Paris, France; ^3^School of Biological Sciences, The University of Hong Kong, Hong Kong, Hong Kong, SAR China; ^4^Boston Children's Hospital, Harvard Medical School, Boston, MA, United States; ^5^State Key Laboratory of Agrobiotechnology, College of Biological Sciences, China Agricultural University, Beijing, China

**Keywords:** DNA replication, replication stress, fork reversal, DNA damage repair, genome instability, cell fate, diseases

Faithful genome duplication through DNA replication is pivotal for genome maintenance. A variety of stresses could challenge this fundamental process and therefore endanger the genome integrity and change the cell fate. These stresses include misincorporation of ribonucleotides, unusual DNA structures, common fragile sites, replication-transcription conflicts, oncogene activation, treatment of pharmacological drugs, and so forth. Replication stress, if not dealt with timely and properly, may result in replication fork collapse, DNA double-strand breaks, and genome instability. Moreover, mutations in replication machinery or factors that combat replication stress can lead to numerous diseases such as developmental defects, microcephaly, anemia, aging, and cancer. Despite intensive studies, how cells deal with replication stress remains poorly understood. Thus, it is of importance to obtain mechanistic insights into DNA replication process and how DNA replication stresses manipulate and determine the cell fate. In this Research Topic, investigators have provided insightful points from their original research or review articles on the source, cellular response, and consequence of replication stress.

## Chapter 1: DNA Replication and Replication Stress

The core of the replisome at a replication fork is the replicative helicase known as CMG (Cdc45-MCM-GINS), which unwinds the double-strand DNA to allow replication. The CMG needs to be unloaded from DNA upon the completion of DNA replication. Xia summarized the recent progress in the field of DNA replication termination, focusing on the mechanisms of CMG disassembly, particularly the ubiquitination factors in yeast and metazoans (Xia). Replication forks often encounter endogenous and exogenous stresses that impede their progression. Tremblay et al. investigated the mechanism of how bacterial virulence factor cytolethal distending toxin (CDT) leads to genetic instability in human cell lines and colorectal organoids from healthy patients' biopsies. They demonstrated that CDT holotoxin induces replicative stress and results in the expression of fragile sites and genome instability. Because some CDT-carrying bacteria were detected in patients with colorectal cancer, the authors proposed that these bacteria could be important therapeutic targets (Tremblay et al.). Next, Lin et al. reported that administration of high doses of eCG (equine chorionic gonadotropin) for *in vitro* fertilization in mice causes ROS (reactive oxygen species), DNA damage and mitotic catastrophe. Lastly, Luo et al. reported that Stattic, a STAT3 inhibitor, induces apoptosis and inhibits proliferation of MV4-11 leukemia cells. Interestingly, the DNA damage repair (DDR) related mRNAs are altered by Sattic treatment, underlying the importance of blocking DNA damage repair (Luo et al.).

## Chapter 2: Fork Processing and Remodeling Under Replication Stress

If stalled forks are not properly processed and restarted, these unstable structures tend to collapse and rearrange. Técher and Pasero reviewed the mechanisms such as fork resection, fork reversal, and mitotic cleavage that ensure the completion of DNA replication under endogenous or exogenous stresses. For the pathological consequences, the processing of stalled forks may release small DNA fragments into the cytoplasm, activating the cGAS-STING pathway and inflammatory response that has both positive and negative impacts on the fate of stressed cells (Técher and Pasero). Moreover, replication fork reversal is a critical protective mechanism in higher eukaryotic cells in response to replication stress. Qiu et al. summarized the key factors and mechanisms required to remodel and protect stalled replication forks. Li et al. provided insights on how, in parallel with the Fanconi anemia pathway, PCNA interactions, and its ubiquitination induced by ICL (inter-strand crosslinks) regulate the recruitment, substrate specificity, activity, and coordinated action of certain nucleases and TLS (translesion DNA synthesis) polymerases during ICL repair and bypass. In addition, increasing numbers of evidence show that protein cofactors are needed for DNA metabolism. Shi et al. outlined the synthesis of mitochondrial, cytosolic and nuclear Iron–sulfur (Fe/S) clusters (ISCs) and discussed the role of ISCs in regulating DNA replication, damage repair and genome integrity.

## Chapter 3: Replication Stress and Diseases

Replication stress and genome instability are hallmarks of cancers and many other diseases. Replication stress is generally considered to be the driving force behind cancer progression. Zhang et al. reported that RECQ1, a DNA helicase critical for fork restart after replication stress, acts at replication forks, binds PCNA, inhibits single-strand DNA formation and nascent strand degradation in glioblastoma cells. In parallel, the structure-specific endonuclease MUS81 plays a vital role in processing the stalled replication forks, and has a close relationship with cancers. Chen et al. reviewed the current understanding of how MUS81 functions in tumors with distinct genetic backgrounds and discussed the potential therapeutic strategies targeting MUS81 in cancer. In addition, HER2-enriched breast carcinomas display evidence of elevated levels of DNA damage associated with replication stress. Liang et al. reported that MiR-92b-3p suppresses proliferation of HER2-positive breast cancer cells by targeting the circular RNA circCDYL. Both circCDYL and miR-92b-3p might be biomarkers in predicting clinical outcomes of HER2-positive breast cancer patients (Liang et al.). Next, DDR and apoptosis are reported to be involved in the pathogenesis of many neurodegenerative diseases such as Spinocerebellar ataxia type 3 (SCA3) and Huntington's disease (HD). The polyQ expansion in ATX3 seems to affect its physiological functions in these distinct pathways. Tu et al. gave a comprehensive overview of the current studies about the physiological roles of ATX3 in DDR and related apoptosis, highlighting the association between these pathways and the pathogenesis of SCA3. Last, high Polθ-mediated end joining (TMEJ, also known as alternative end-joining) activity was observed in the breast cancer subgroup deficient of HR (homologous recombination). Prodhomme et al. discussed their recent finding that EMT (epithelial-to-mesenchymal transition) transcription factor ZEB1 modulates TMEJ activity by direct transcriptional suppression, providing insights into the relationship between replication stress and TMEJ or EMT features, and how these processes contribute to genomic stability.

Taken together, the articles in this issue could help researchers better understand the sources of replication stress and their cellular consequences, especially their impact on genome integrity, cell viability, and human diseases.

## Author Contributions

LD wrote the manuscript. HL edited the manuscript. All authors provided intellectual input to the editorial.

## Funding

LD was supported by the Guangdong Basic and Applied Basic Research Foundation (2020A1515110542). C-LC was supported by the YPI program of I. Curie, the ATIP-Avenir program from Center national de la recherche scientifique (CNRS) and Plan Cancer from INSERM, the CNRS 80|Prime interdisciplinary program, the Agence Nationale pour la Recherche (ANR) and Institut National du Cancer (INCa). YZ was supported by the University of Hong Kong and the Research Grants Council (RGC) of Hong Kong (GRF16104617, GRF16103918, and C7009-20GF). YD was supported by American Heart Association (AHA) Grant (12SDG8760002). HL was supported by the National Key R&D Program of China (2019YFA0903900); National Natural Science Foundation of China Grants (31630005 and 31770084).

## Conflict of Interest

The authors declare that the research was conducted in the absence of any commercial or financial relationships that could be construed as a potential conflict of interest.

## Publisher's Note

All claims expressed in this article are solely those of the authors and do not necessarily represent those of their affiliated organizations, or those of the publisher, the editors and the reviewers. Any product that may be evaluated in this article, or claim that may be made by its manufacturer, is not guaranteed or endorsed by the publisher.

